# Supplementary material for: Brain activity regulates loose coupling between mitochondrial and cytosolic Ca2+ transients
Source: Nat Commun. 2019 Nov 21;10:5277. doi: 10.1038/s41467-019-13142-0 (PMC6872662; doi:10.1038/s41467-019-13142-0)
Supplement: Supplementary file 1 — Supplementary Information [file 41467_2019_13142_MOESM1_ESM.pdf]

Nature Communications

Supplementary information

Brain Activity Regulates Loose Coupling between Mitochondrial and Cytosolic Ca^{2+} Transients

Yuan Lin *et.al*

This PDF file includes:
Supplementary figure 1-8

Supplementary figure 1

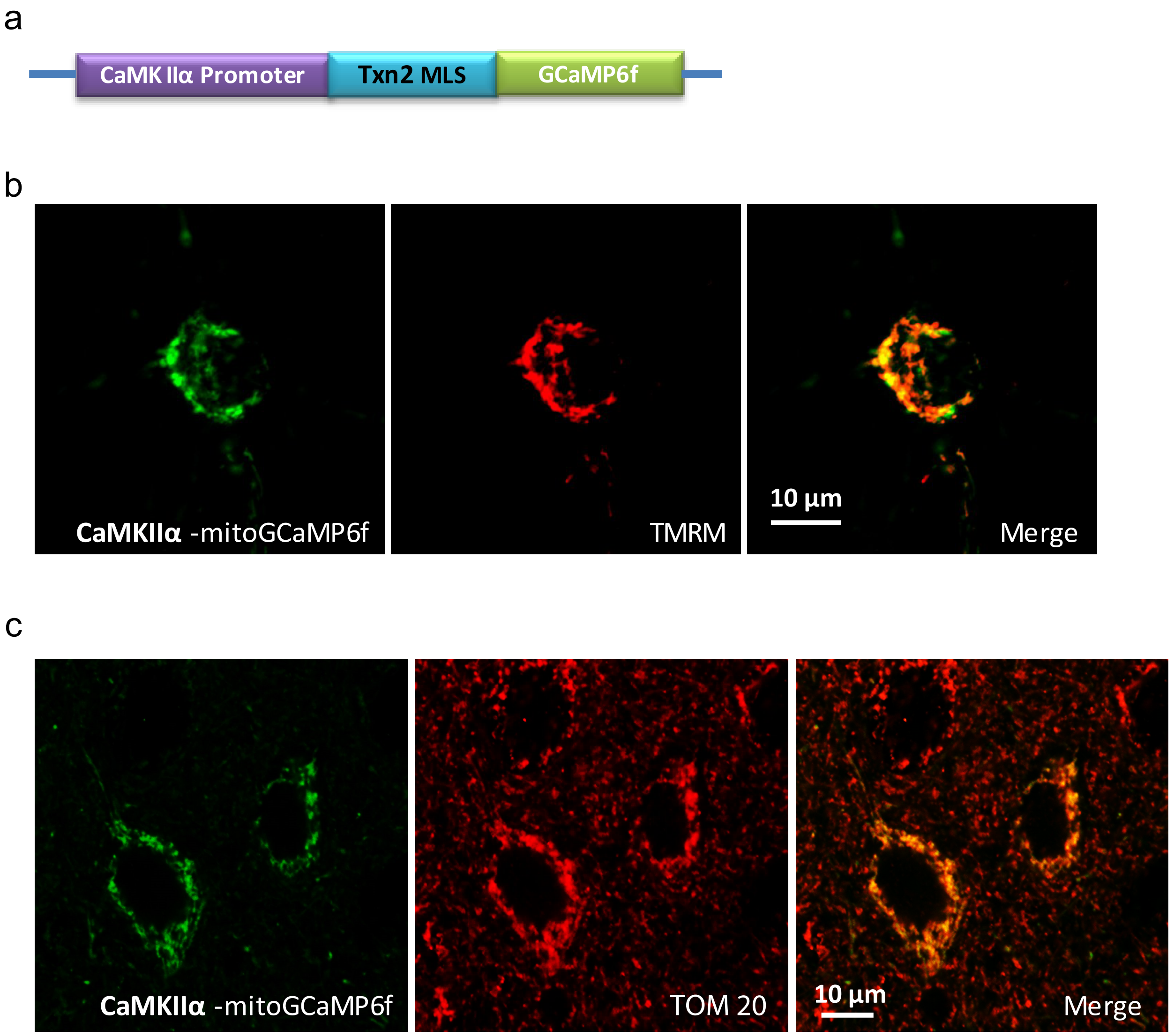

Supplementary Figure 1. Mitochondrial targeting of GCaMP6f in neurons. ***a*** Schematic of the viral construct. Purple box: Ca^{2+}/calmodulin-dependent protein kinase type II subunit alpha (CaMK IIα) promoter. Blue box: Mitochondrial targeting sequences of thioredoxin 2 gene. Green box: genetically encoded Ca^{2+} indicator GCaMP6f. ***b*** Representative confocal images of TMRM and mito-GCaMP6f staining of cultured neonatal mouse cortical neurons. ***c*** Immunohistochemical staining showing co-localization of mitochondrial GCaMP6f and mitochondrial outer-membrane marker TOM20 in brain slice.

Supplementary figure 2

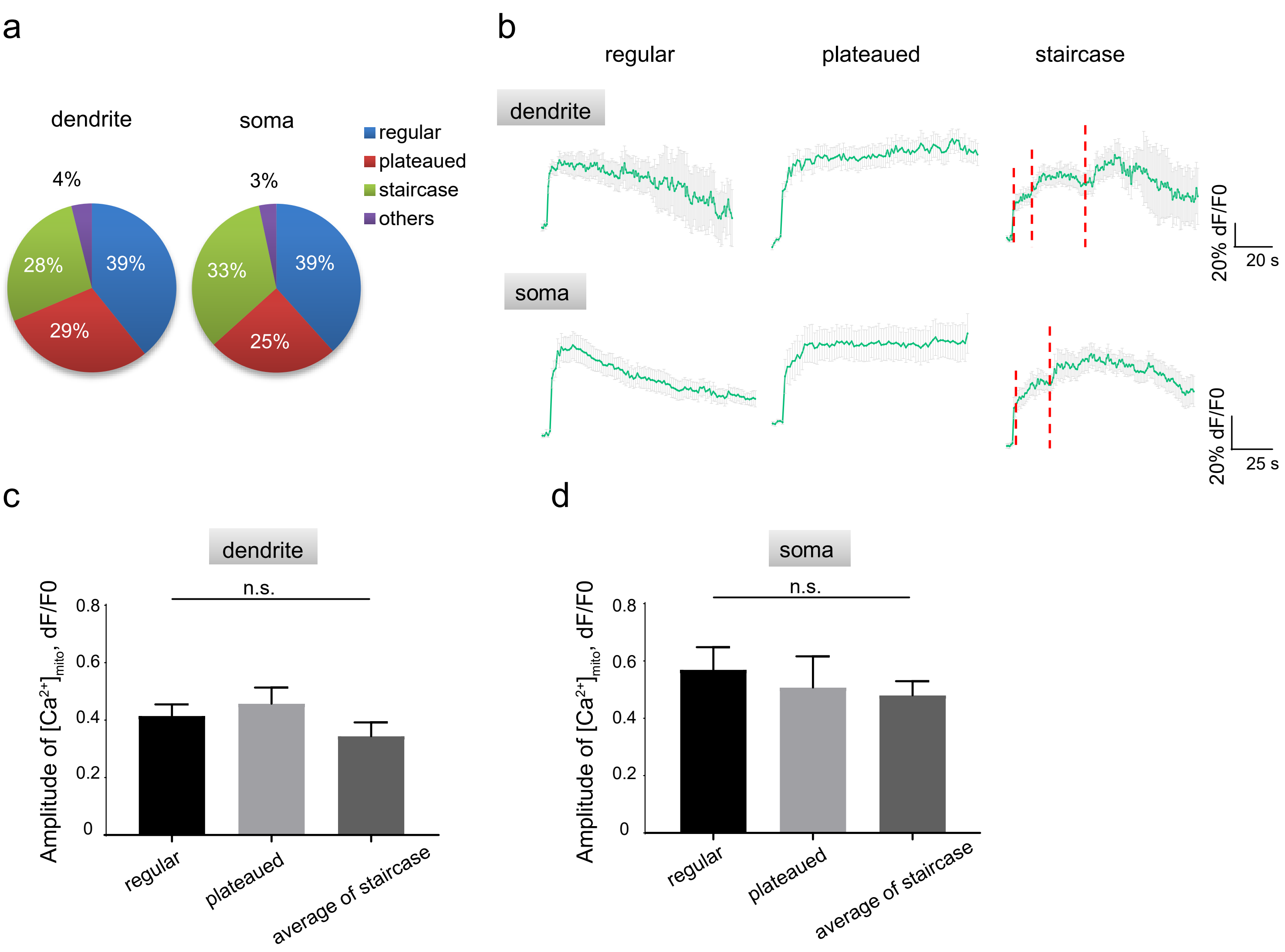

Supplementary figure 2. Three types of $[Ca^{2+}]_{mito}$ transients.
a Percent of three main types of $[Ca^{2+}]_{mito}$ transients for data obtained during treadmill running in dendrites (left, n = 50 events from 6 mice) and somas (right, n = 64 events from 9 mice). ***b*** Averaged $[Ca^{2+}]_{mito}$ transients for the regular, staircase, and plateaue types in dendrites (upper panel) and somas (lower panel) during running. 12-16 traces used for each group. ***c-d*** Amplitudes of different types of $[Ca^{2+}]_{mito}$ transients in dendrites (c, n = 12-16 for each group) and somas (d, n = 11-15 for each group). Data are presented as mean ± SEM. **P* < 0.05. ***P* < 0.01. ****P* < 0.0001, one way ANOVA and multiple comparisons. Source data are provided as a Source Data file.

Supplementary figure 3

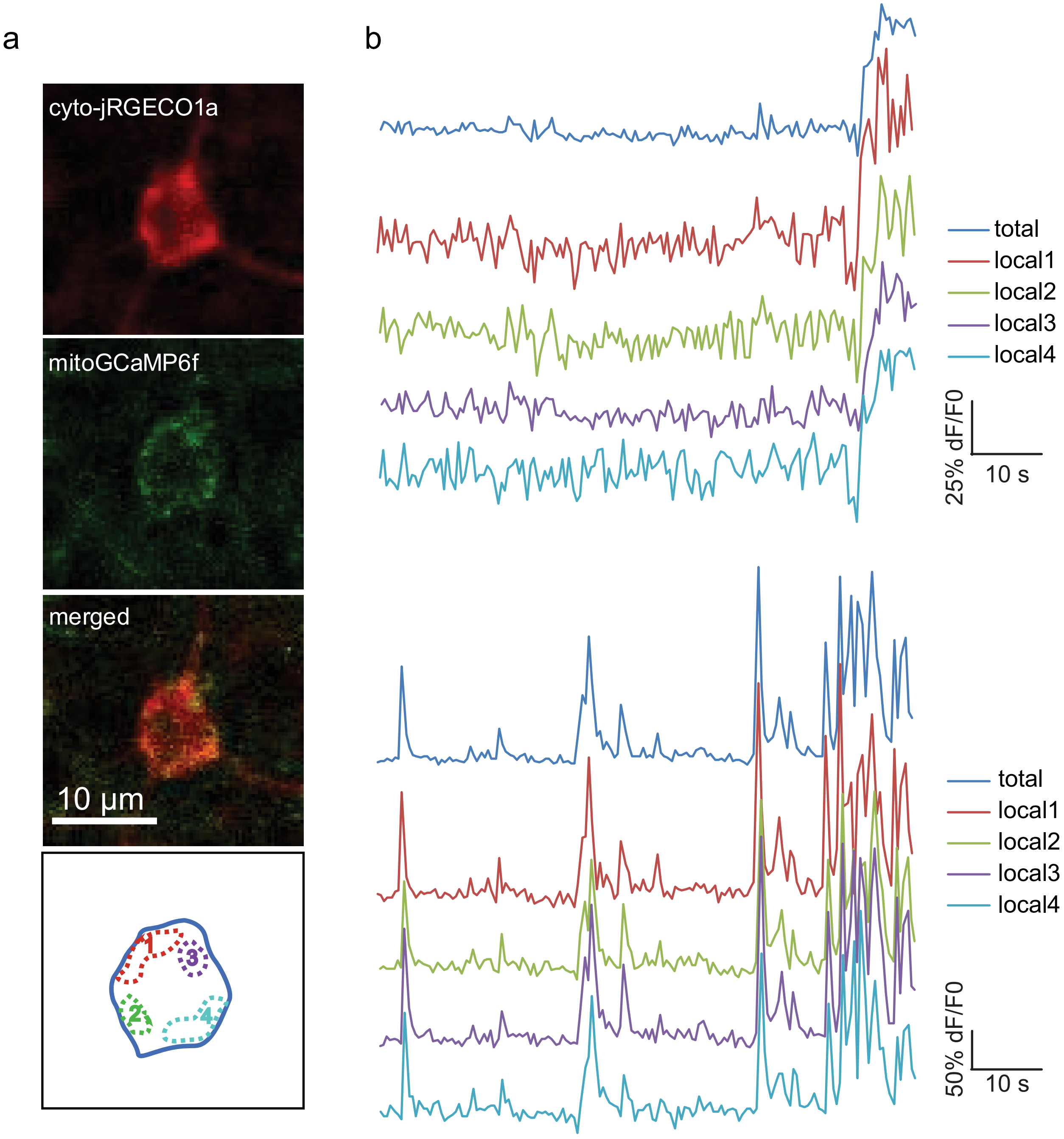

Supplementary Figure 3. An example showing spatially synchronized $[Ca^{2+}]_{cyto}$ and $[Ca^{2+}]_{mito}$ transients at different locations in a soma.

a Representative images of double labeling in a soma and the corresponding schematic of different regions of interest. ***b*** Time courses of fluorescence changes of $[Ca^{2+}]_{mito}$ (upper) and $[Ca^{2+}]_{cyto}$ (lower) activities at different locations as shown in the schematic on the left panel.

Supplementary figure 4

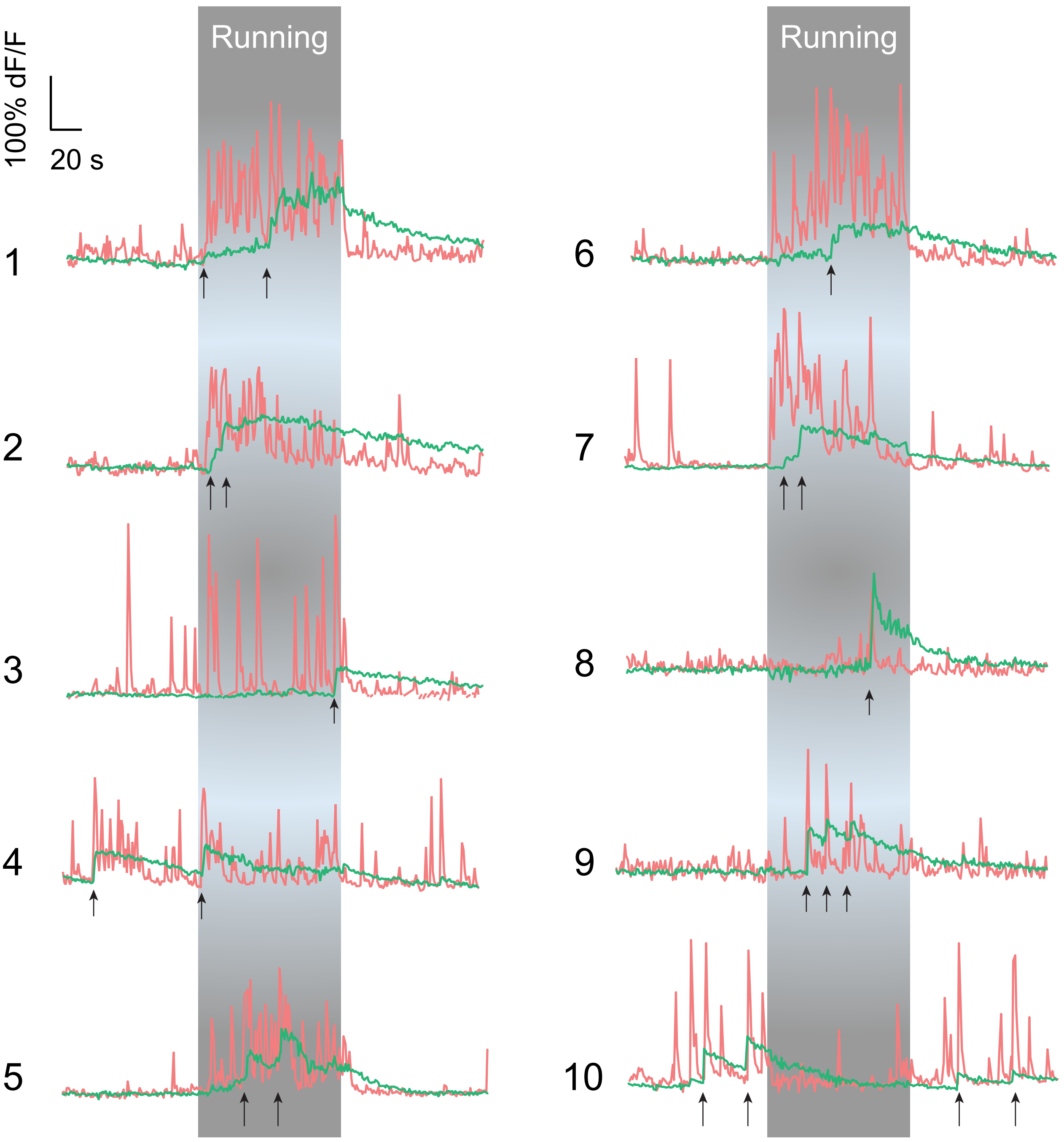

Supplementary figure 4. Patterns of $[Ca^{2+}]_{mito}$-to-$[Ca^{2+}]_{cyto}$ coupling and uncoupling in somas of M1 neurons before, during and after running.

Note the complex kinetics or "computation" for a $[Ca^{2+}]_{cyto}$ transient (in red color) to trigger (or not to trigger) a $[Ca^{2+}]_{mito}$ transient (in green color).Note the sharp rises of $[Ca^{2+}]_{mito}$ transients (marked with arrows) and the variable latencies of the $[Ca^{2+}]_{mito}$-to-$[Ca^{2+}]_{cyto}$ coupling .

Supplementary figure 5

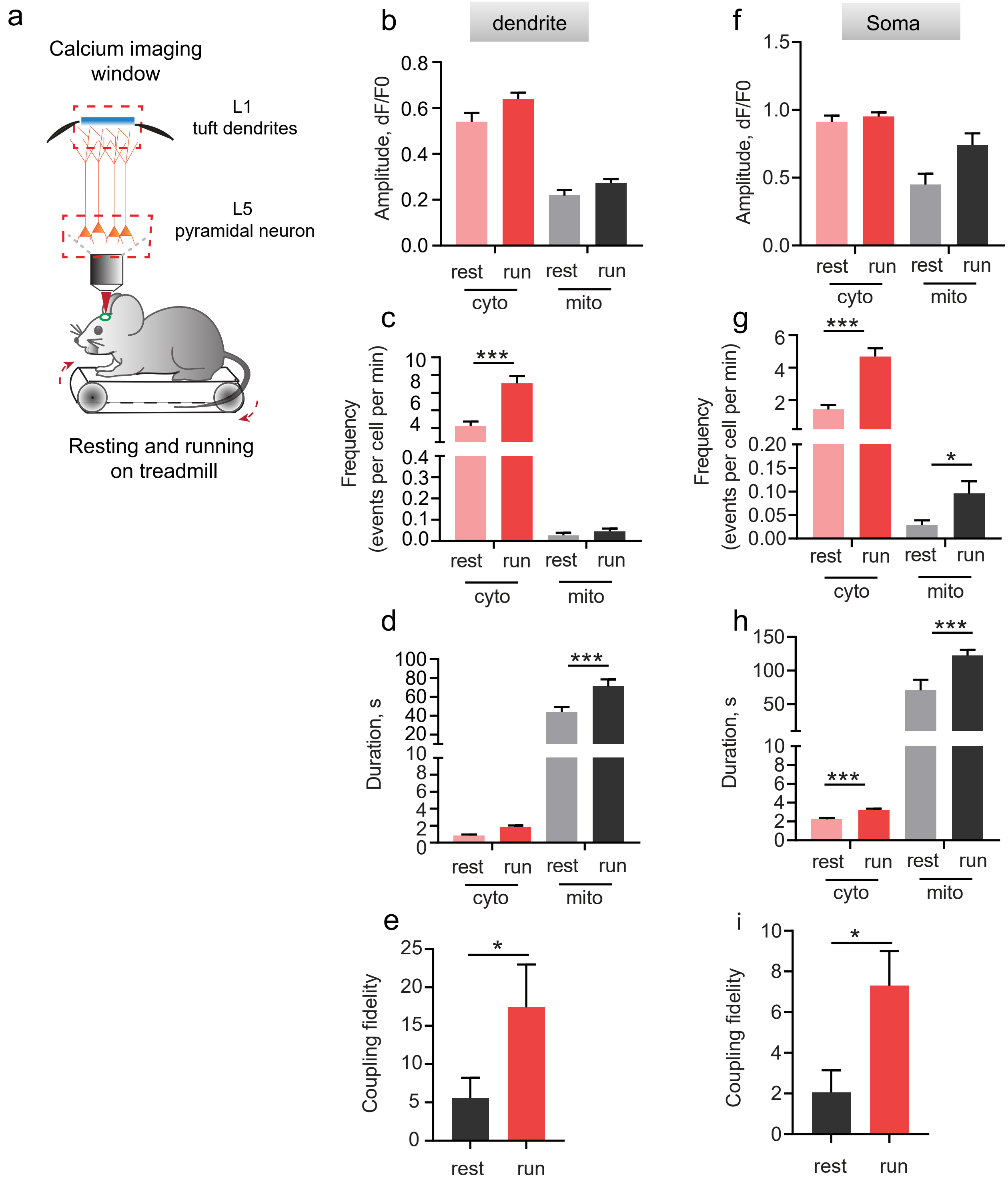

Supplementary figure 5. Exercise enhanced $[Ca^{2+}]_{mito}$-to-$[Ca^{2+}]_{cyto}$ coupling in L5 neurons in the forelimb motor cortex.

a Schematic of transcranial two-photon imaging in L5 pyramidal neurons (~ 500 μm deep from pial surface) and their projected dendrites in L1 in the forelimb motor cortex of awake, head-restrained mice. ***b-d*** Amplitudes, frequencies and duration of $[Ca^{2+}]_{cyto}$ and $[Ca^{2+}]_{mito}$ transients in dendrites (n = 7-100 from 4 mice). ***h-g*** Amplitudes, frequencies and duration of $[Ca^{2+}]_{cyto}$ and $[Ca^{2+}]_{mito}$ transients somas (n = 5-256 from 4 mice) at rest and during treadmill running. ***e and i***, Coupling fidelity at rest and during running in dendrites (e, n = 17-27 events from 4 mice) and somas (i, n = 30-34 events from 4 mice). Data are presented as mean ± SEM. *$P < 0.05$. **$P < 0.01$. ***$P < 0.0001$, unpaired T test. Source data are provided as a Source Data file.

Supplementary figure 6

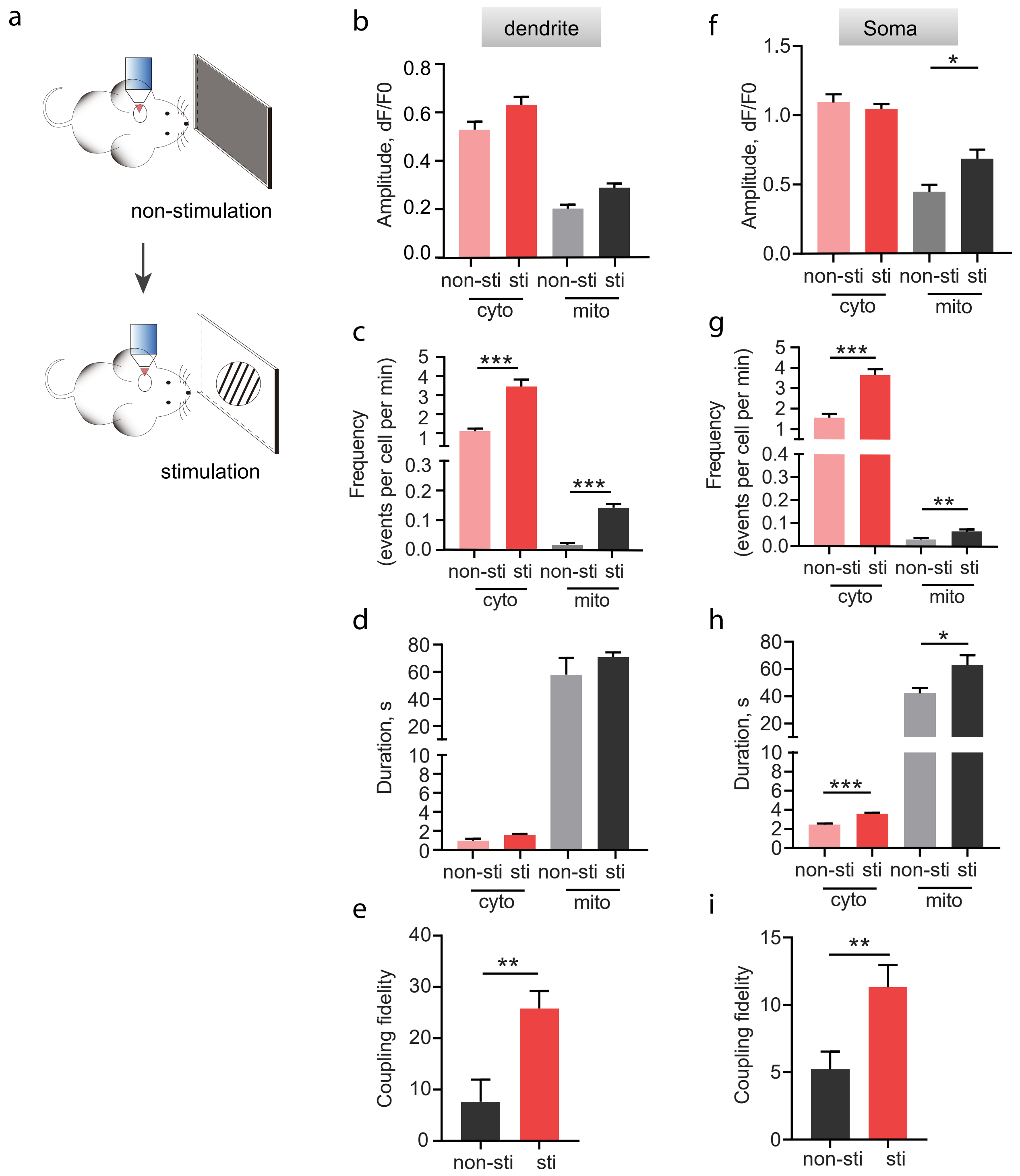

Supplementary figure 6. Visual stimulation enhanced $[Ca^{2+}]_{mito}$-to-$[Ca^{2+}]_{cyto}$ coupling in L2/3 neurons of primary visual cortex.

a Schematic of transcranial two-photon imaging in L2/3 neurons of the primary visual cortex in awake behaving mice with or without visual stimulation. ***b-d*** Amplitudes, frequencies and duration of $[Ca^{2+}]_{cyto}$ and $[Ca^{2+}]_{mito}$ transients in dendrites (n = 5-100 from 8 mice). ***h-g*** Amplitudes, frequencies and duration of $[Ca^{2+}]_{cyto}$ and $[Ca^{2+}]_{mito}$ transients somas (n = 5-256 from 8 mice) at rest and during treadmill running. ***e and i***, Coupling fidelity before and after visual stimulation in dendrites (e, n = 26-35 events from 8 mice) and somas (i, n = 57-58 events from 8 mice). Data are presented as mean ± SEM. *P < 0.05. **P < 0.01. ***P < 0.0001, unpaired T test. Source data are provided as a Source Data file.

Supplementary figure 7

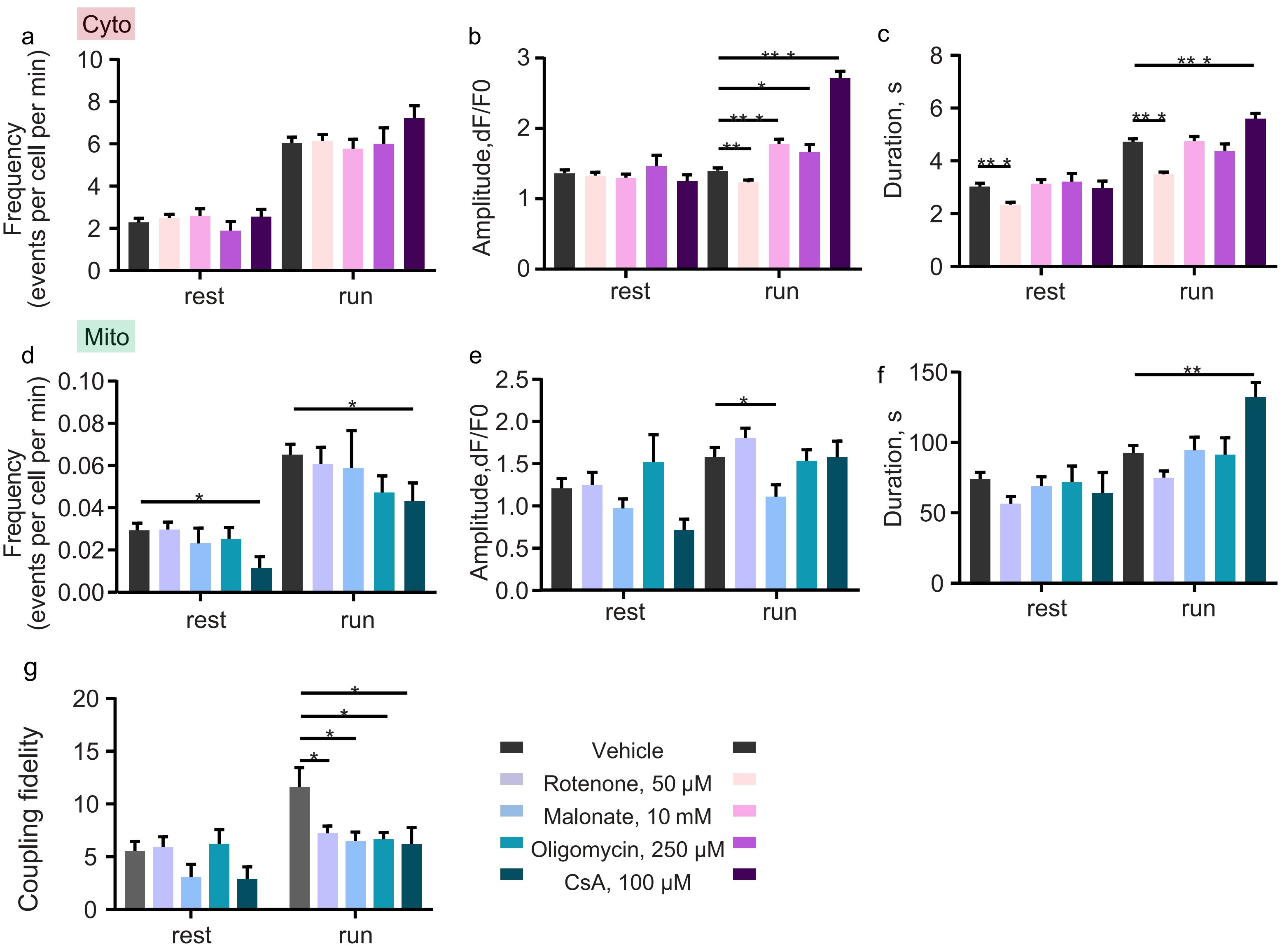

Supplementary figure 7. Effects of ETC and MPTP inhibitors on $[Ca^{2+}]_{mito}$-to-$[Ca^{2+}]_{cyto}$ coupling

a-f Effects of ETC and MPTP inhibitors on somatic $[Ca^{2+}]_{cyto}$ (**a-c**) and $[Ca^{2+}]_{mito}$ (**d-f**) transient frequency, amplitude, duration in L2/3 M1 neurons. n = 4-6 mice. 1 μl of 50 μM Rotenone, 10 μM Malonate, 250 μM Oligomycin, 100 μM CsA in ACSF, or ACSF alone was injected locally in L2/3 and left to settle for 10 minutes before imaging. ***g*** All ETC and MPTP inhibitors used significantly decreased somatic $[Ca^{2+}]_{mito}$-to-$[Ca^{2+}]_{cyto}$ coupling fidelity during running. **P* < 0.05. ***P* < 0.01. ****P* < 0.0001, by one-way ANOVA and multiple comparisons. Source data are provided as a Source Data file.

Supplementary figure 8

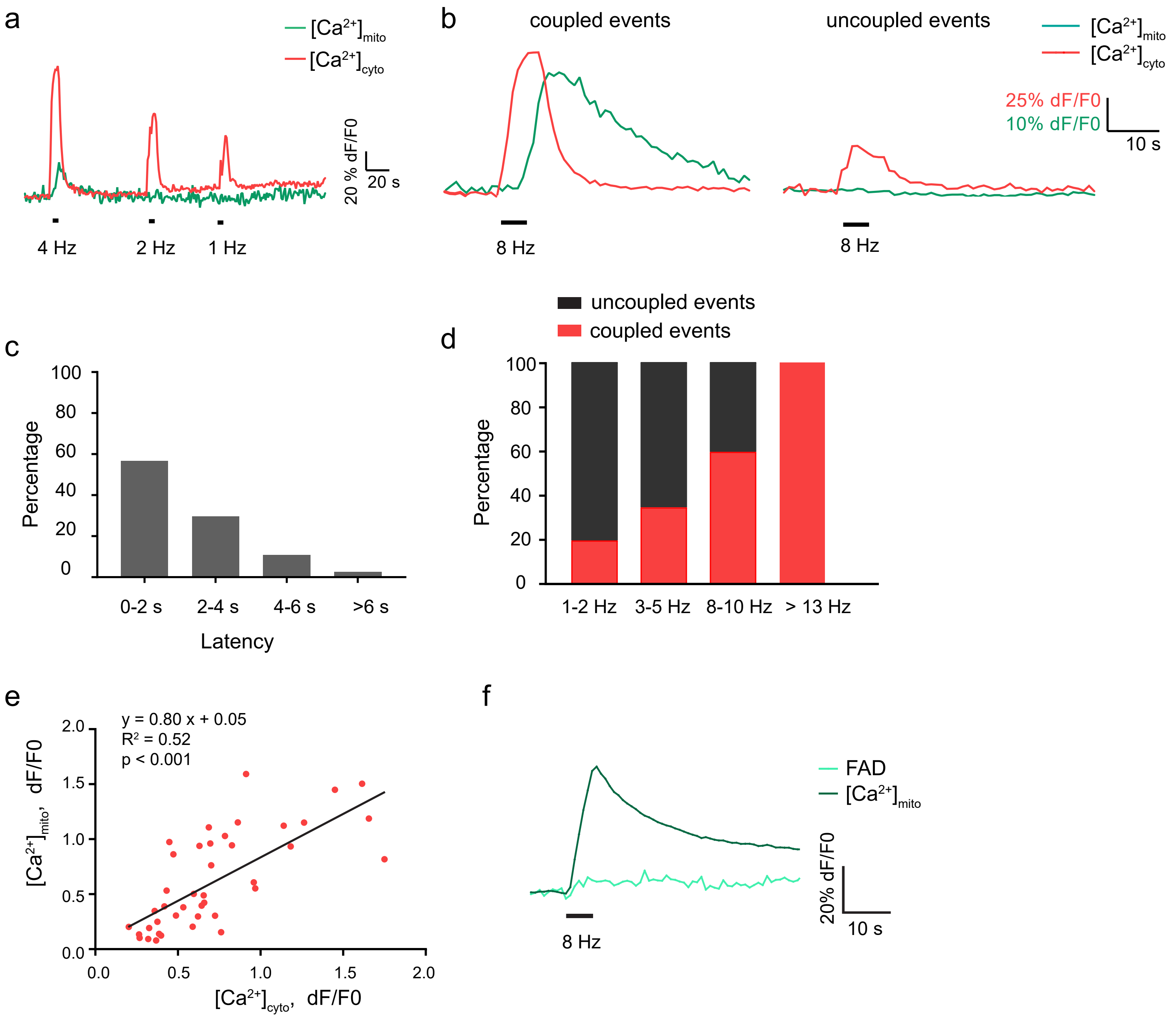

Supplementary figure 8. $[Ca^{2+}]_{mito}$-to-$[Ca^{2+}]_{cyto}$ coupling in cultured neonatal mouse cortial neurons.

a Representative example showing somatic $[Ca^{2+}]_{mito}$-to-$[Ca^{2+}]_{cyto}$ coupling and uncoupling in cultured neonatal mouse cortical neurons responding to field electrical stimulation. ***b*** Averaged traces of coupled (left panel) and uncoupled pairs of $[Ca^{2+}]_{cyto}$ and $[Ca^{2+}]_{mito}$ transients (right panel). 8 traces were used for each group. ***c*** Latency of somatic $[Ca^{2+}]_{mito}$-to-$[Ca^{2+}]_{cyto}$ coupling (n= 37 paired events in 15 cells). ***d*** Dependence of the coupling fidelity on electrical field stimulation frequency. (1-2 Hz, n= 26; 3-5 Hz, n= 38; 8-10 Hz, n= 32; >13 Hz, n= 13). ***e*** Correlation of amplitudes of $[Ca^{2+}]_{cyto}$ and $[Ca^{2+}]_{mito}$ transients for coupled events (n= 41 paired events in 18 cells). ***f*** Averaged traces of $[Ca^{2+}]_{mito}$ transients and corresponding FAD autofluorescence. 17 traces were used for each group. Source data are provided as a Source Data file.
