# Supplementary material for: Brain activity regulates loose coupling between mitochondrial and cytosolic Ca2+ transients
Source: Nat Commun. 2019 Nov 21;10:5277. doi: 10.1038/s41467-019-13142-0 (PMC6872662; doi:10.1038/s41467-019-13142-0)
Supplement: Supplementary file 4 — Description of Additional Supplementary Files [file 41467_2019_13142_MOESM4_ESM.pdf]

Description of Additional Supplementary Files

File Name: Supplementary Movie 1
Description: In vivo two-photon imaging of $[Ca^{2+}]_{mito}$ and $[Ca^{2+}]_{cyto}$ transients in L1 dendrites. The region of image was at ~ 30 µm below the pia in primary motor cortex. The white arrow indicates a $[Ca^{2+}]_{mito}$-to-$[Ca^{2+}]_{cyto}$ coupling event.

File Name: Supplementary Movie 2
Description: In vivo two-photon imaging of $[Ca^{2+}]_{mito}$ and $[Ca^{2+}]_{cyto}$ transients in L2/3 somas. The region of image was at ~ 300 µm below the pia in primary motor cortex. The time window that mouse was running on the treadmill was labeled as "RUN" on upper left corner. The white arrows indicate representative $[Ca^{2+}]_{mito}$-to-$[Ca^{2+}]_{cyto}$ coupling events.

File Name: Supplementary Movie 3
Description: No pH change detected by mito-pHtomato either at rest or during running. The region of image was at ~ 30 µm below the pia in primary motor cortex
